# Evaluation of Two Osmosis-Based Methods for the Preparation of Drug Delivery Systems Based on Red Blood Cells

**DOI:** 10.3390/pharmaceutics15092281

**Published:** 2023-09-05

**Authors:** Carmen Gutierrez-Millan, Celia Barez Diaz, Lydia Alvarez Vizan, Clara I. Colino

**Affiliations:** 1 Area of Pharmacy and Pharmaceutical Technology, Department of Pharmaceutical Sciences, University of Salamanca, 37007 Salamanca, Spain; 2 Institute for Biomedical Research of Salamanca (IBSAL), 37007 Salamanca, Spain

**Keywords:** loaded erythrocytes, hypotonic dilution, drug delivery, hypotonic dialysis, osmosis-based loading methods

## Abstract

Erythrocytes have been thoroughly investigated as drug delivery systems for a wide range of therapeutic molecules and using different kinds of loading methods, outstanding the osmosis-based methods as the most used ones. Most of them involve too much handling of blood components and the immediate obtention of fresh blood. Based on our group’s considerable experience in dialysis-based carrier erythrocyte preparation, this study details a simple method based on hypotonic dilution and subsequent resealing that has been developed for stavudine using packed erythrocytes from a local blood bank. Properties of the obtained carrier erythrocytes were studied in comparison to those prepared by dialysis. Erythrocytes’ morphology, osmotic fragility, hematological parameters, and in vitro release profiles were evaluated. Loaded erythrocytes obtained with the proposed method did not show impaired properties in comparison with those obtained with our reference method, provided that the buffer composition remained the same. In the present work, we have optimized a simplified method for erythrocytes’ drug loading, which can use blood transfusion products and could be easily automatized and scalable.

## 1. Introduction

Since the 70s to nowadays, erythrocytes have been explored for their application as vehicles of different foreign molecules [1,2,3,4]. Their versatility, ubiquity, biocompatibility, and biodegradability make them an excellent alternative for the vectorization of drugs, proteins, genetic material, or other kinds of markers and diagnostic tools [5,6].

The shape, membrane fragility, flexibility, and hematological indices are essential erythrocyte parameters. However, during the cargo process, they may undergo irreversible changes, causing erythrocytes to be eliminated more rapidly from the bloodstream. Those properties, together with the intrinsic characteristics of the charged molecule, lead to important differences in its encapsulation efficiency and release profile [7,8].

Throughout this time, different erythrocyte loading methods have been proposed, from electroporation to cell-penetrating peptide-mediated ones [9,10,11]. Osmosis-based methods are currently the most standardized strategies for encapsulating substances [12]. Among this approach, there are several variants, such as hypotonic dilution, osmotic pulse, hypotonic hemolysis, and hypotonic dialysis, which differ in the methodology itself, but all are based on subjecting the erythrocytes to a hypotonic solution, thus achieving the opening of pores in the membrane to allow the drug entrance. Through these pores, drugs that otherwise do not enter the cells can penetrate into the erythrocytes. In the last stages, the membrane is repaired with an iso or hypertonic medium, and the drug remains entrapped inside. In the hypotonic dialysis, erythrocytes are separated from the medium by a semipermeable membrane, whereas in the hypotonic dilution method, the erythrocytes are directly mixed first with the hypotonic medium and then with the hypertonic one.

The loading methods applied in clinics are mainly based on hypotonic dialysis and need specific devices or facilities [12]. Hypotonic dilution-based methods are easier to develop and involve less erythrocyte handling. Although these two kinds of methods have been studied for carrier erythrocyte preparation, information about the comparison of hypotonic dilution and dialysis methods is scarce in the recent literature. From our experience with dialysis-based methods of loading erythrocytes from different species for use as drug delivery systems [13,14], we want to explore the hypotonic dilution method to evaluate the feasibility of this procedure for stavudine encapsulation. With this aim, we have optimized a new osmosis-based method with hypotonic dilution that may be performed with the basic equipment commonly available in any laboratory. This method may lead to the simplification of experimental procedures. Considering that these systems are prepared in a personalized way, these will facilitate clinical translation and widen the erythrocytes’ applications.

Stavudine is a nucleoside reverse transcriptase inhibitor used as an antiretroviral drug that has a very short half-life and high water solubility [14]. This work aims to design ghost erythrocytes as biocompatible and biodegradable carriers for the delivery of stavudine within a combinational approach for treating HIV infections. It is a well-known problem that antiretroviral drugs do not reach the reservoirs where the virus remains and constitute the main cause that prevents HIV eradication. Thus, it is necessary to develop alternative strategies that can combat the AIDS disease. Its inclusion in drug delivery systems has been previously studied with the objective of improving biodistribution and combatting the intracellular reservoirs that hamper HIV eradication [15]. Ghost erythrocytes allow the drug’s vectorization to macrophages [16] and also constitute a controlled drug delivery system that may improve pharmacokinetic stavudine disposition. 

Besides the simplification of the loading procedure, in this work, we propose to use erythrocytes from the blood bank. Although the most common origin of the native erythrocytes in this kind of procedure is fresh autologous erythrocytes [14,17,18], the use of cells from a blood bank could widen the applicability and automatization of loaded erythrocyte preparation.

## 2. Materials and Methods

### 2.1. Loading Methods

**Figure 1:**
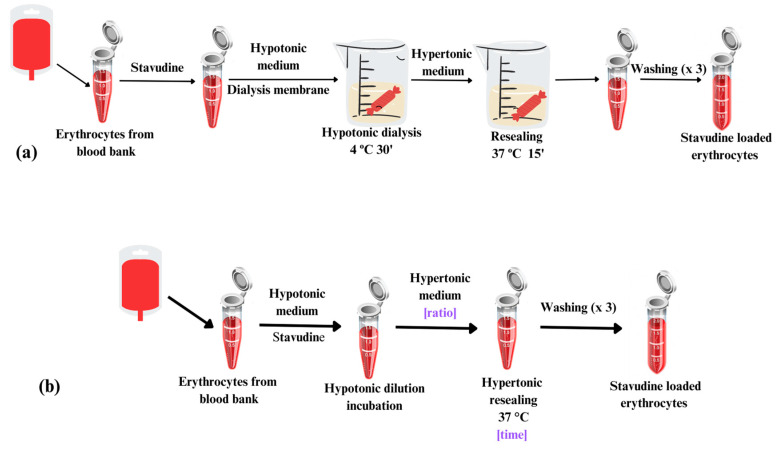
Main steps of the two studied encapsulation methods (**a**) Hypotonic dialysis method; (**b**) Hypotonic dilution method.

Different osmosis-based methods have been proved (Figure 1). Loaded erythrocytes obtained from our previous classical method have been used as the reference group. Thus, the encapsulation of stavudine in this group was accomplished using an optimization of our previously described hypotonic dialysis method [19] for stavudine encapsulation. Packed erythrocytes from local blood bank were resuspended in Hanks-PBS with stavudine to give a suspension of 70% of packed erythrocytes. This cell suspension containing stavudine was placed in a dialysis bag (Medicell, molecular size cut-off, 12–14 KDa), and dialysis was performed in a ratio 1:30 (*v*:*v*) against a hypotonic buffer with osmolality of ~90 mOsm/kg for 30 min at 4 °C. Resealing was achieved by subsequent incubation against 30 mL of hypertonic (~500 mOsm/Kg) buffer for 15 min at 37 °C. Resealed erythrocytes were washed twice with isotonic Hanks-PBS buffer in order to remove the non-entrapped drug.

Regarding the hypotonic dilution method, a suspension of 70% (*v*:*v*) of packed erythrocytes is prepared in hypotonic buffer containing the drug and incubated at 4 °C. Next, hypertonic buffer is added to this mixture, and a resealing step is performed at 37 °C in order to restore the tonicity and incubation. Final washings with isotonic medium are also included to remove the non-entrapped drug excess.

Previous studies developed by our group let us establish 30 min as the optimum time for hypotonic incubation in order to maximize the stavudine loading efficacy in human blood bank erythrocytes. 

Once the conditions for this first step were established, and considering resealing as one of the most critical stages to avoid drug loss, studies focused on optimizing the resealing stage. Two factors were studied: the ratio volume of erythrocyte dispersion:hypertonic buffer (*v*:*v*), and the time for resealing, as shown in Table 1. 

For both encapsulation methods, loaded erythrocytes are counted by optical microscopy with a Neubauer chamber in order to standardize the encapsulated amount of drug with the number of erythrocytes. 

In the next step, going further with the aim of simplifying the encapsulation procedure, the hypotonic medium composition was modified to avoid complex macromolecules inclusion. Based on previous studies, our usual hypotonic buffer (15 mM NaH_2_PO_4_·2H_2_O, 15 mM NaHCO_3_, 20 mM glucose, 2 mM ATP, 3 mM glutathione reduced, and 5 mM NaCl, pH 7.4, ~90 mOsm/kg) was replaced by 5-mmol/L KH_2_PO_4_, 5-mmol/L K_2_HPO_4_ (pH 7.4, ~20 mOsm/kg) (conditions N_c_), and the loaded erythrocytes obtained were characterized and compared with the results obtained with the usual hypotonic buffer.

### 2.2. Drug Encapsulation

For estimation of drug encapsulation, loaded erythrocytes were centrifuged (Centrifuge 5418R Eppendorf (Hamburg, Germany) at 10,900 rpm for 5 min, and stavudine was quantified in the loaded erythrocytes by UFLC. Results of drug encapsulation are expressed as drug concentration estimated in loaded erythrocytes and also standardized by the number of cells counted for each individual experience.

Encapsulation efficiency (EE) is calculated as the percentage of the total available drug that is entrapped inside, as follows:

EE = Drug in loaded erythrocytes × 100/Initial available drug(1)

### 2.3. Stavudine Quantification

Stavudine was determined using a UFLC chromatograph (UFLCXR Shimadzu, Kyoto, Japan) equipped with a PDA SPD-M20 detector and a C18 Kinetex^®^ column (Phenomenex^®^ 50 mm × 2.10 mm, 1.7 µm). The mobile phase was an aqueous solution of 0.1% formic acid and acetonitrile (97:3) with a flow rate of 0.50 mL/min at 45 °C. Detection wavelength was 265 nm. Sample treatment was carried out by precipitation with 30% trichloroacetic acid (TCA) in a 10:1 ratio (sample:TCA) and filtration through a pore size of 0.22 µm prior to analysis.

### 2.4. Osmotic Fragility

The osmotic fragility of native erythrocytes and stavudine-loaded carrier erythrocytes was determined according to Dacie’s method as described elsewhere [20]. Carrier erythrocytes were incubated in the presence of various concentrations of NaCl (0% to 0.9%), and the released hemoglobin was measured at 418 nm (spectrophotometer 8453 Agilent, Santa Clara, CA, USA). Osmotic fragility curves were fitted using GraphPad Prism software (San Diego, CA, USA), and an osmotic fragility index was defined for native and stavudine-loaded erythrocytes. This index was calculated as the NaCl concentration (%) necessary for obtaining 50% of hemolysis according to the curve equation.

### 2.5. Hematological Parameters

Standard hematological parameters in the control and loaded erythrocytes, such as hematocrit (HCT), hemoglobin (HGB), total red blood cell count (RBCS), red cell distribution width (RDW), mean corpuscular volume (MCV) mean corpuscular hemoglobin (MCH), and mean corpuscular hemoglobin concentration (MCHC), were estimated by spectrophotometry and impedance analysis using a Horiba Medical’s Yumizen hematology autoanalyzer (Kyoto, Japan). 

### 2.6. Microscopic Analysis

Packed erythrocytes were fixed with glutaraldehyde and then postfixed in osmium tetroxide. After ethanol dehydration with a graded ethanol series, samples were critical point-dried and sputter-coated with gold. 

Images obtained with a Jeol IT500 Scanning Electron Microscope (Akishima, Tokyo, Japan) were analyzed for a qualitative evaluation of changes in morphology, and quantitative analysis of erythrocyte sizes was performed with ImageJ software version 1.53 (Bethesda, MD, USA).

### 2.7. In Vitro Release

In vitro release was performed by including the loaded erythrocytes in a regenerated cellulose semipermeable membrane (MWCO 12,000–14,000 Da, pore diameter of 25 Å). In order to guarantee the sink conditions, 300 µL of stavudine carrier erythrocytes were incubated for 24 h at 37 °C in 3 mL of isotonic medium (thermostated bath UNITRONIC, OR Selecta, Cham, Switzerland). Aliquots were removed at predefined times (5 min to 24 h), and the drug released was analytically determined by UFLC. 

### 2.8. Statistical Analysis

For the statistical analysis of the encapsulation results and hematological parameters, analysis of variance was performed using SPSS statistical software version 28.0.1.1 (Chicago, IL, USA). The differences were considered statistically significant when *p* < 0.05.

## 3. Results and Discussion

In the present work, we have compared dialysis and hypotonic dilution methods for stavudine encapsulation in ghost erythrocytes in order to confirm the potential of the simpler hypotonic dilution procedure. The influence of the resealing conditions and the hypotonic buffer composition in the key parameters of the carrier erythrocytes were studied.

### 3.1. Optimization of Resealing Conditions

#### 3.1.1. Stavudine Encapsulation

The results for drug encapsulation are compiled in Table 2.

Regarding the hypotonic dilution methods, ANOVA results reflect statistically significant differences in the encapsulated concentration in the diverse conditions studied for both the time factor and the ratio factor (*v*:*v*), and the interaction between the two is also significant (*p* < 0.001). 

Table 2 shows higher concentration values estimated for C conditions, but if we observe the efficiency values, the results among the resealing conditions tested are more similar, and no statistically significant differences were found, although a slightly higher value was observed for C, with 32.86%. It is when we standardize the encapsulation results by cell count that the difference becomes more apparent. 

Although hypotonic dilution methods are reported to lead to lower entrapment efficiency [8], our work shows better results for this parameter with the hypotonic dilution method in comparison with the dialysis method, no matter which resealing conditions we assess. When the encapsulated amount is normalized by cell count, the values of dialysis-loaded erythrocytes are in the same range as those obtained with conditions B and D. Conditions A lead to the worst encapsulation results, which indicates that time and ratio applied in these conditions are insufficient for stavudine loading.

Concentration and efficiency figures seem to point out that C conditions give us better results compared to other resealing conditions regarding the hypotonic dilution method. Since 15 min resealing seems to be insufficient, and 30 min has also been proposed by other authors [18,20], we consider it as a convenient time that does not excessively extend the loading procedure.

#### 3.1.2. Osmotic Fragility

**Figure 2:**
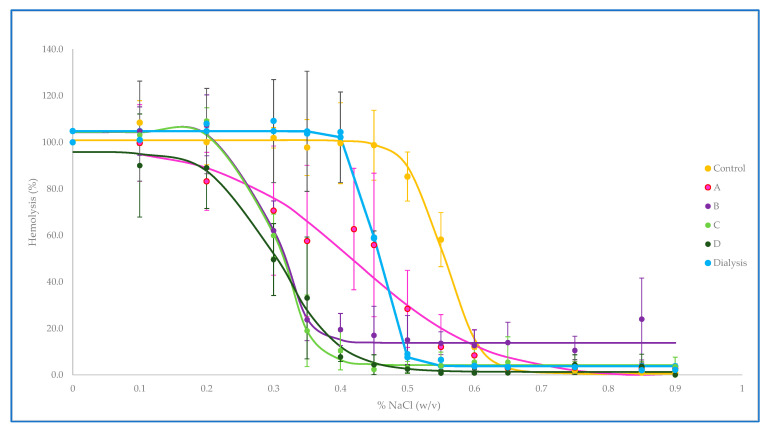
Control and loaded erythrocytes osmotic fragility curves (A, B, C, and D are loaded erythrocytes with hypotonic dilution method with the different resealing conditions). The figure shows the hemolysis measured (mean ± S.D.) when exposed to different NaCl concentrations. The percentage of hemolysis is estimated from the signal obtained from incubation with pure water (100% hemolysis).

The osmotic fragility curves reflect the hemolysis suffered by the loaded erythrocytes when exposed to different osmolalities as an indirect estimate of the alteration of their properties with respect to control erythrocytes. Despite the fact that a high loss of hemoglobin has been pointed out as one of the main drawbacks of hypotonic dilution methods [8], we have not obtained curves far different from the erythrocytes loaded by dialysis, as it has also been described by other authors [18]. All the conditions to which the erythrocytes have been subjected in this study cause them to undergo similar variations, with a shift of the curves to the left compared to control erythrocytes, as can be seen in Figure 2. Procedure A is the one in which the morphology of the curve is most altered with respect to the control, which could indicate that the resealing conditions in A are too mild to achieve full recovery of the original membrane properties.

The values of the osmotic fragility index, shown in Table 3, indicate the NaCl concentration at which 50% hemolysis occurs. The lower the osmotic fragility index, the lower the osmolality of the NaCl concentration that causes hemolysis of half of the erythrocyte population. Our results, which show lower index values for loaded erythrocytes, may seem paradoxical because they imply a greater resistance to hemolysis in the loaded erythrocytes than in the original ones, but they are concordant to those previously obtained in our group [14] and do not differ too much from the conclusions of other authors who have obtained values close to those of native erythrocytes [14,20]. This fact could be attributed to the removal of the more fragile erythrocytes in the final washes included in the encapsulation procedure. 

Our results show the importance of full curve characterization and not only the osmotic fragility index because while the A estimated osmotic fragility index is the closest to that of control and dialysis-loaded erythrocytes, the visual analysis of the curve shape leads us to different conclusions about the potential damage of loaded erythrocytes.

#### 3.1.3. Hematological Parameters

Table 4 shows the hematological parameters of control and erythrocytes loaded with the different conditions.

The main parameters related to the properties of the erythrocytes showed similar values for all the conditions studied. Although we can observe some differences among control erythrocytes and loaded ones, treatments do not induce drastic changes in these properties. Values are close either for those loaded by hypotonic dilution or by dialysis, a treatment that has been demonstrated to involve an adequate behavior of loaded erythrocytes when reinjected in experimental animals [19].

The most affected parameters are those related to the number of cells, such as hematocrit and erythrocyte total count, but considering that in the experimental procedure, we fix the volume ratio between packed erythrocytes and hypo or hyper buffers, these differences could be attributed more to the error associated with the volume measurements than to the alterations induced in the loaded erythrocytes because of the drug loading.

Despite having subjected the cells to a hypotonic medium, hemoglobin content-related parameters seem not to have been seriously affected. The size of treated erythrocytes is also recovered after the treatment, as reflected by the VCM of loaded erythrocytes compared to the values of the control group. Even more, hypotonic dilution-based loading procedures induced lower heterogeneity in erythrocyte populations than the dialysis-based one, as indicated by the values observed for RDW.

Analysis of variance confirmed that there are no statistically significant differences (*p* > 0.05) in most of the parameters studied, except for RDW, MCV, and HCM, which are significantly affected by the resealing ratio. 

We observe that our new hypotonic dilution method does not induce detrimental effects on hematological parameters compared to the erythrocytes loaded with dialysis. Although the hypotonic dilution could initially be perceived as a more aggressive method than the dialysis-based one, our results question this assumption. This could be attributed to the use of the same hypotonic buffer as in the dialysis procedure and to the final osmolality of the mixture of hypotonic and hypertonic medium to which erythrocytes are exposed in our procedure. 

#### 3.1.4. Morphology

**Figure 3:**
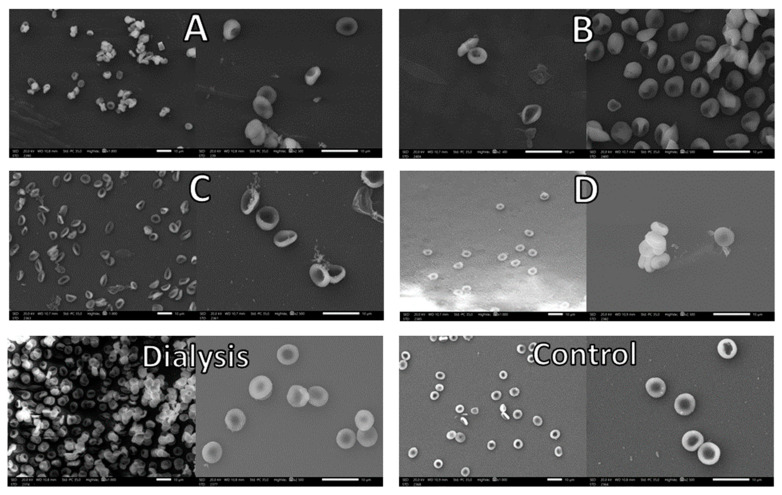
SEM images of control and loaded erythrocytes obtained by dialysis and different hypotonic dilution variations (**A**–**D**). For each condition, a general field (on the **left**) and specific details (on the **right**) are shown.

Micrographs of control and loaded erythrocytes obtained after the different loading conditions are shown in Figure 3.

A first glimpse let us observe that A and B conditions induce an apparently more swollen morphology in loaded erythrocytes, suggesting that the increase of the resealing time followed in conditions C and D leads to a better restoration of the physiological conditions after drug encapsulation, recovering the characteristic biconcave shape of erythrocytes to a great extent in a similar way to our previous reference procedure (dialysis-based method), as it can be seen in the corresponding figure.

After quantitative analysis, measurements of the sizes of loaded and control erythrocytes confirmed (Table 5) that the most important differences are observed in the A group with respect to the control, suggesting that either a shorter time or a lower volume of hypertonic medium leads to worse resealing after loading.

#### 3.1.5. In Vitro Release

**Figure 4:**
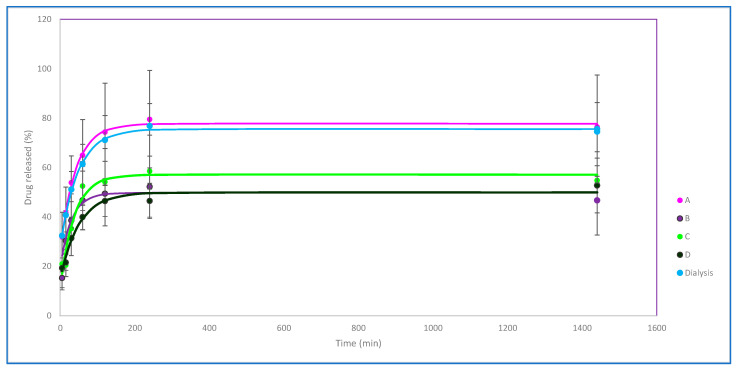
In vitro release profiles of loaded erythrocytes obtained with the different methods and conditions studied (curves A, B, C, and D correspond to loaded erythrocytes with hypotonic dilution method with the different resealing conditions). Mean ± S.D. values of percentage of released drug are represented for each time.

Stavudine release studies from hypotonic dilution-loaded erythrocytes showed similar profiles to the previous reference method based on hypotonic dialysis (Figure 4). Regarding the different conditions of hypotonic dilution methods, the release stabilizes in all of them after 4 h and maintains the levels for 24 h. 

The A conditions, which combine the least resealing time and hypertonic buffer volume, result in a higher release rate and a high maximum of almost 80% in the period studied, which is reached at 6 h. These results are consistent with other results already observed for these conditions, while the curve profiles observed for B, C, and D are similar, maintaining about 50% of the encapsulated drug inside throughout the entire interval studied.

According to these profiles, it could be suggested that a better degree of erythrocyte resealing could affect the drug release rate and that the proposed hypotonic dilution method even seems to prevent drug leakage better than the previous reference method.

### 3.2. Comparative Analysis of Simplified Hypoosmotic Buffer Results

Considering that previous results pointed out C conditions as the preferential resealing procedure, the simplified hypoosmotic buffer was tested by using a resealing of 30 min with a 1:0.5 erythrocyte dispersion:hypertonic buffer (*v*:*v*) ratio (conditions N_c_).

Regarding stavudine encapsulation, the simplified buffer led to a lower drug concentration (0.20 ± 0.08 mg/mL) and efficiency (9.22 ± 3.94%), but when standardized by cell count, we can observe higher values (7.49 ± 0.21 mg/×10^10^ cells) than those obtained for C conditions.

**Figure 5:**
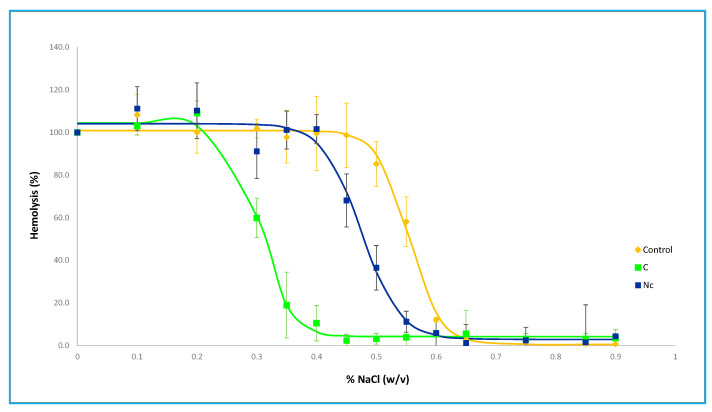
Control and loaded erythrocytes with conditions C and N_c_ osmotic fragility curves. The figure shows the hemolysis measured (mean ± S.D.) when exposed to different NaCl concentrations. Percentage of hemolysis is estimated from the signal obtained from incubation with pure water (100% hemolysis).

Analysis of the profiles of the curves (Figure 5) shows that erythrocytes loaded with N_c_ conditions have a morphology closer to control erythrocytes than to C conditions. The left shift of the curve is intermediate between control and C conditions.

Consequently, the osmotic fragility index estimated for N_c_ erythrocytes is 0.48, also between control and C erythrocytes. This value is consistent with other loaded erythrocyte indices mentioned above, which show values lower than the control group.

Comparative statistical analysis of hematological parameters observed for N_c_ group versus C conditions (data summary in Table 6) revealed significant differences (*p* < 0.05) for all the studied parameters, except for RBCS count (number/µL), RDW and CHCM which could be attributed to the excessively low osmolality of this hypotonic buffer.

**Figure 6:**
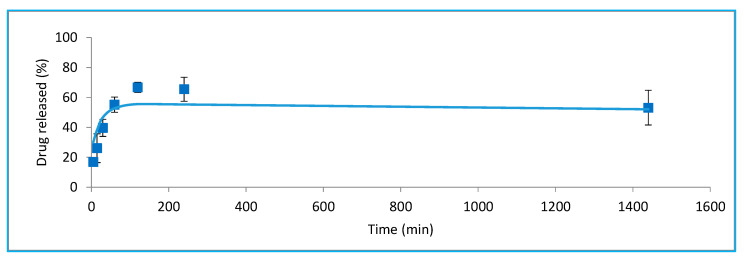
In vitro release profiles of loaded erythrocytes obtained with N_C_ conditions. Mean ± S.D. values of percentage of released drug are represented for each time.

In vitro release assays from N_c_ conditions-loaded erythrocytes also showed similar profiles to C-loaded ones, providing sustained drug levels, maintaining around half of the drug encapsulated inside them for 24 h (Figure 6).

In summary, these preliminary results obtained for erythrocytes loaded with N_c_ conditions showed differences with the C conditions previously studied, such as some important erythrocytes hematological parameters (MCV and HCM), whereas other features, such as release profiles, are maintained.

It seems that the use of a simpler loading buffer with relevant lower values of osmolality could not conveniently preserve the loaded erythrocytes’ properties, although the new conditions should be more thoroughly explored to confirm this assumption.

## 4. Conclusions

In this work, taking our standard dialysis-based loaded erythrocytes procedure as a starting point and reference, we have compared and optimized a hypotonic dilution method for stavudine encapsulation. 

Results showed that erythrocytes obtained present suitable properties for their use as drug delivery systems with the resealing conditions of 30 min and the ratio volume of erythrocyte dispersion: hypertonic buffer (*v*:*v*) of 1:0.5 being the most adequate among those studied. 

Moreover, we have used packed erythrocytes provided by a local blood bank in all the procedures studied, thus confirming the feasibility of using this kind of product as the source for preparing loaded erythrocytes as drug delivery systems.

The stavudine ghost erythrocytes developed constitute a controlled release drug delivery system that may prolong the plasma residence time of stavudine and increase its access to macrophage reservoirs, improving the success of the fight against AIDS infection.

Preliminary assays for the use of a simpler hypotonic medium to avoid the use of macromolecules have been carried out. In view of the obtained results, this alternative needs to be studied more in-depth.

In summary, we have demonstrated that loaded erythrocytes can be obtained from blood transfusion products with a really easy-to-perform method that implies minor handling. The use of transfusion products as raw material and the simplicity of the newly proposed method would increase the successful automation of these carrier systems’ obtention and allow compliance with quality requirements, which broadens the horizons of applicability of these systems and their clinical translation.

## Figures and Tables

**Table 1:** Resealing conditions assayed for stavudine encapsulation in erythrocytes by hypotonic dilution method.

Resealing Conditions	Ratio (*v*:*v*)	Time (min)
A	1:0.5	15
B	1:1.5	15
C	1:0.5	30
D	1:1.5	30

**Table 2:** Stavudine encapsulation results for the different procedures and conditions assayed. * Significant differences (*p* < 0.001).

	Concentration (mg/mL) *		Efficiency (%)		Encapsulated Drug per Cell Number (mg/×10^10^ Cells)	
	Mean	SD	Mean	SD	Mean	SD
A	0.28	0.10	26.11	9.73	0.33	0.20
B	0.28	0.14	24.81	14.67	0.68	0.16
C	0.63	0.16	32.86	12.60	1.64	0.41
D	0.29	0.12	29.80	8.49	0.60	0.17
Dialysis	0.40	0.22	19.04	7.17	0.66	0.59

**Table 3:** Osmotic fragility indexes of control and loaded erythrocytes.

Control	A	B	C	D	Dialysis
0.55	0.42	0.31	0.31	0.30	0.46

**Table 4:** Main hematological parameters (mean ± S.D.) of control and loaded erythrocytes. * Significant differences (*p* < 0.05).

Control	Control Erythrocytes	A	B	C	D	Dialysis
Hematocrit (%)	47.23 ± 3.45	63.73 ± 2.50	69.53 ± 2.25	69.27 ± 0.64	66.73 ± 6.71	62.00 ± 5.24
HGB (g/dL)	15.63 ± 0.92	21.53 ± 1.03	23.53 ± 1.33	23.27 ± 0.64	22.4 ± 2.90	20.20 ± 1.91
RBCS (number/μL)	5.12 × 10^6^ ±4.17 × 10^5^	7.11 × 10^6^ ±2.91 × 10^5^	7.79 × 10^6^ ±3.19 × 10^5^	7.69 × 10^6^ ±1.86 × 10^5^	7.47 × 10^6^ ±7.91 × 10^5^	7.21 × 10^6^ ±5.34 × 10^5^
RDW *	13.70 ± 0.26	13.40 ± 0.50	13.13 ± 0.12	13.47 ± 0.93	13.40 ± 0.30	17.40 ± 0.70
MCV (fL) *	92.30 ± 1.42	89.69 ± 0.32	89.32 ± 0.76	90.14 ± 1.33	89.33 ± 0.48	85.99 ± 1.24
HCM (pg) *	30.55 ± 1.14	30.29 ± 0.27	30.21 ± 0.47	30.27 ± 0.23	30.02 ± 0.73	28.00 ± 0.73
CHCM (g/dL)	33.10 ± 0.80	33.78 ± 0.42	33.83 ± 0.81	33.59 ± 0.64	33.60 ± 0.99	32.57 ± 0.95

**Table 5:** Sizes (nm) of control and loaded erythrocytes.

Control	A	B	C	D	Dialysis
6.64 ± 0.45	5.59 ± 0.61	6.29 ± 0.52	6.68 ± 0.69	5.99 ± 0.53	6.00 ± 0.36

**Table 6:** Main hematological parameters (mean ± S.D.) for control erythrocytes, C, and N_c_ conditions. * Significant differences (*p* < 0.05).

Control	Control Erythrocytes	C	N_c_
Hematocrit (%) *	47.23 ± 3.45	69.27 ± 0.64	65.00 ± 1.261
HGB (g/dL) *	15.63 ± 0.92	23.27 ± 0.64	21.50 ± 0.38
RBCS (number/µL)	5.12 × 10^6^ ±4.17 × 10^5^	7.69 × 10^6^ ±1.86 × 10^5^	7.74 × 10^6^ ±1.45 × 10^5^
RDW	13.70 ± 0.26	13.47 ± 0.93	16.45 ± 0.33
VCM (fL) *	92.30 ± 1.42	90.14 ± 1.33	84.03 ± 0.06
HCM (pg) *	30.55 ± 1.14	30.27 ± 0.23	27.80 ± 0.06
CHCM (g/dL)	33.10 ± 0.80	33.59 ± 0.64	33.08 ± 010

## References

[1] Sun Y., Su J., Liu G., Chen J., Zhang X., Zhang R., Jiang M., Qiu M. (2017). Advances of blood cell-based drug delivery systems. Eur. J. Pharm. Sci..

[2] Wang S., Han K., Ma S., Qi X., Guo L., Li X. (2022). Blood cells as supercarrier systems for advanced drug delivery. Med. Drug Discov..

[3] Dong H., Tu T., Jiang Y., Yuan Y., Peng F., Deng Y., Ren C., Liu C. (2023). Erythrocyte-based Drug Delivery: How Far from Clinical Application?. Curr. Drug Deliv..

[4] Rossi L., Pierigè F., Aliano M.P., Magnani M. (2020). Ongoing Developments and Clinical Progress in Drug-Loaded Red Blood Cell Technologies. BioDrugs.

[5] Millán C.G., Castañeda A.Z., Marinero M.L.S., Lanao J.M. (2004). Factors associated with the performance of carrier erythrocytes obtained by hypotonic dialysis. Blood Cells Mol. Dis..

[6] Koleva L., Bovt E., Ataullakhanov F., Sinauridze E. (2020). Erythrocytes as Carriers: From Drug Delivery to Biosensors. Pharmaceutics.

[7] López S.C.B., Meissner K.E. (2017). Characterization of carrier erythrocytes for biosensing applications. J. Biomed. Opt..

[8] Hamidi M., Tajerzadeh H. (2003). Carrier Erythrocytes: An Overview. Drug Deliv..

[9] Tan S., Wu T., Zhang D., Zhang Z. (2015). Cell or Cell Membrane-Based Drug Delivery Systems. Theranostics.

[10] He H., Ye J., Wang Y., Liu Q., Chung H.S., Kwon Y.M., Shin M.C., Lee K., Yang V.C. (2014). Cell-penetrating peptides meditated encapsulation of protein therapeutics into intact red blood cells and its application. J. Control. Release.

[11] Rao L., Cai B., Bu L.-L., Liao Q.-Q., Guo S.-S., Zhao X.-Z., Dong W.-F., Liu W. (2017). Microfluidic Electroporation-Facilitated Synthesis of Erythrocyte Membrane-Coated Magnetic Nanoparticles for Enhanced Imaging-Guided Cancer Therapy. ACS Nano.

[12] Li Y., Raza F., Liu Y., Wei Y., Rong R., Zheng M., Yuan W., Su J., Qiu M. (2021). Clinical progress and advanced research of red blood cells based drug delivery system. Biomaterials.

[13] Millán C.G., Castañeda A.Z., López F.G., Marinero M.L.S., Lanao J.M., Arévalo M. (2005). Encapsulation and In Vitro Evaluation of Amikacin-Loaded Erythrocytes. Drug Deliv..

[14] Millán C.G., Bax B.E., Castañeda A.Z., Marinero M.L.S., Lanao J.M. (2008). In vitro studies of amikacin-loaded human carrier erythrocytes. Transl. Res..

[15] Zazo H., Colino C.I., Gutiérrez-Millán C., Cordero A.A., Bartneck M., Lanao J.M. (2022). Physiologically Based Pharmacokinetic (PBPK) Model of Gold Nanoparticle-Based Drug Delivery System for Stavudine Biodistribution. Pharmaceutics.

[16] Briones E., Colino C.I., Millán C.G., Lanao J.M. (2009). Increasing the selectivity of amikacin in rat peritoneal macrophages using carrier erythrocytes. Eur. J. Pharm. Sci..

[17] Coker S.A., Szczepiorkowski Z.M., Siegel A.H., Ferrari A., Mambrini G., Anand R., Hartman R.D., Benatti L., Dumont L.J. (2018). A Study of the Pharmacokinetic Properties and the In Vivo Kinetics of Erythrocytes Loaded with Dexamethasone Sodium Phosphate in Healthy Volunteers. Transfus. Med. Rev..

[18] Zhang X., Qiu M., Guo P., Lian Y., Xu E., Su J. (2018). Autologous Red Blood Cell Delivery of Betamethasone Phosphate Sodium for Long Anti-Inflammation. Pharmaceutics.

[19] Millán C.G., Castañeda A.Z., López F.G., Marinero M.L.S., Lanao J.M. (2008). Pharmacokinetics and biodistribution of amikacin encapsulated in carrier erythrocytes. J. Antimicrob. Chemother..

[20] Robert M., Laperrousaz B., Piedrahita D., Gautier E.-F., Nemkov T., Dupuy F., Nader E., Salnot V., Mayeux P., D’Alessandro A. (2022). Multiparametric characterization of red blood cell physiology after hypotonic dialysis based drug encapsulation process. Acta Pharm. Sin. B.

